# Infection increases vulnerability to climate change via effects on host thermal tolerance

**DOI:** 10.1038/s41598-017-09950-3

**Published:** 2017-08-24

**Authors:** Sasha E. Greenspan, Deborah S. Bower, Elizabeth A. Roznik, David A. Pike, Gerry Marantelli, Ross A. Alford, Lin Schwarzkopf, Brett R. Scheffers

**Affiliations:** 1 College of Science and Engineering, James Cook University, Townsville, Queensland 4811 Australia; 2 Department of Research and Conservation, Memphis Zoo, Memphis, TN 38112 USA; 3 Department of Biology, Rhodes College, Memphis, TN 38112 USA; 4 Amphibian Research Centre, Pearcedale, Victoria 3912 Australia; 5 Department of Wildlife Ecology and Conservation, University of Florida, Gainesville, Florida 32611 USA

## Abstract

Unprecedented global climate change and increasing rates of infectious disease emergence are occurring simultaneously. Infection with emerging pathogens may alter the thermal thresholds of hosts. However, the effects of fungal infection on host thermal limits have not been examined. Moreover, the influence of infections on the heat tolerance of hosts has rarely been investigated within the context of realistic thermal acclimation regimes and potential anthropogenic climate change. We tested for effects of fungal infection on host thermal tolerance in a model system: frogs infected with the chytrid *Batrachochytrium dendrobatidis*. Infection reduced the critical thermal maxima (CT_max_) of hosts by up to ~4 °C. Acclimation to realistic daily heat pulses enhanced thermal tolerance among infected individuals, but the magnitude of the parasitism effect usually exceeded the magnitude of the acclimation effect. In ectotherms, behaviors that elevate body temperature may decrease parasite performance or increase immune function, thereby reducing infection risk or the intensity of existing infections. However, increased heat sensitivity from infections may discourage these protective behaviors, even at temperatures below critical maxima, tipping the balance in favor of the parasite. We conclude that infectious disease could lead to increased uncertainty in estimates of species’ vulnerability to climate change.

## Introduction

Projections of the future global climate indicate that temperature means, variances, and extremes will change^1–6^. These changes may be hazardous for some animals by shifting daily, seasonal, or intermittent temperature cycles away from optimal conditions or closer to lethal extremes^7^. Risks to populations due to climate change can be estimated using warming tolerance, which is the difference between the species’ maximum heat tolerance (critical thermal maximum [CT_max_]) and maximum environmental temperature^8–11^. When this value is large, individuals theoretically have a high thermal safety margin in the context of rising environmental temperatures^11^. In contrast, when this value is small, risk is high because even slight increases in environmental temperatures may cause the body temperatures of individuals to reach lethal limits^12^. This is further compounded when temperatures approaching critical thermal maxima lead to behaviors or ecological interactions that reduce fitness. For example, heat stress may cause individuals to seek refuge at the expense of activities that promote fitness (e.g., foraging or reproduction)^13^. Similarly, altered temperature patterns may lead to changes in phenology, resource availability, or predator interactions that threaten individual and population survival^14^.

Thermal stress and fitness costs associated with global climate change are likely to occur in combination with other natural and anthropogenic stressors such as land use change, environmental contaminants, and disease^15–17^. Fungal diseases are currently emerging at record rates, posing a direct threat to global biodiversity in the face of climate change^18^. Reduced maximum thermal tolerance can be a major side effect of infections in amphibians^19^, fish^20, 21^, and mollusks^22–28^. For example, ill newts *Notophthalmus viridescens* infected with a mesomycetozoan parasite had lower CT_max_ than uninfected newts (by 0.6–1.7 °C)^19^. Similarly, resistance to high temperature (hours at 25 °C until 50% mortality) was lower in brook trout *Salvelinus fontinalis* infested with gill lice *Salmincola edwardsii*, and was inversely correlated with extent of secondary bacterial infection, a measure of fish health^20^. Thus, infections have the potential to synergistically interact with ectotherm physiology to reduce warming tolerance, which could render individuals and populations more vulnerable to rising temperatures from climate change or habitat modification. However, the effect of fungal infection on host thermal limits has not been tested and the influence of infections on host thermal limits has rarely been investigated in the context of realistic, fluctuating thermal acclimation regimes.

Here we investigate interactions between fungal disease and upper thermal tolerance in a model host-pathogen system: frogs infected with the chytrid *Batrachochytrium dendrobatidis* (Bd)^29^. We experimentally infected frogs with Bd and acclimated them to constant cool temperatures or daily heat pulses mimicking the body temperature regimes of frogs in nature. We then examined the effects of Bd infection status, infection intensity and acclimation on their critical thermal maxima and considered the implications of our findings in light of current and projected global change.

## Results

**Figure 1:**
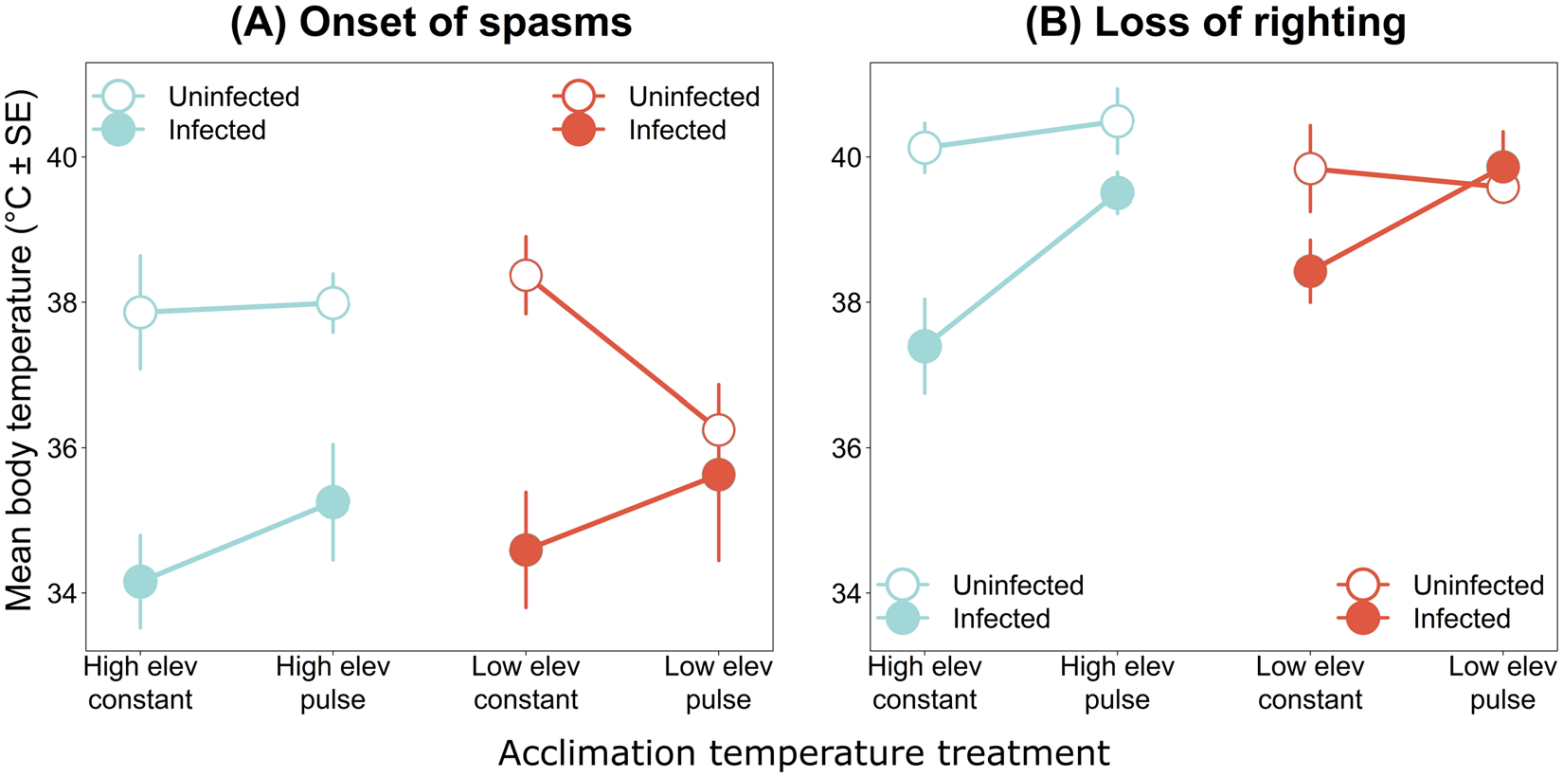
Average critical thermal maxima (± SE) for the model amphibian host *Litoria spenceri* acclimated to four temperature treatments, with and without infections by the fungus *Batrachochytrium dendrobatidis*. Metrics of the critical thermal maximum were (**A**) body temperature at onset of spasms and (**B**) body temperature at loss of righting ability.

The critical thermal maxima of our model frog species *Litoria spenceri*, measured as temperature at onset of spasms and temperature at loss of righting ability, were significantly lower for Bd-infected frogs than for uninfected frogs (spasms: p < 0.001; righting: p = 0.009; Table 1; Fig. 1), after controlling for a positive relationship between frog snout-urostyle length and critical thermal maxima (Table 1). Across acclimation temperature treatments, mean temperature at onset of spasms ( ± SD) ranged from 34.2 °C ± 2.1 °C to 35.6 °C ± 3.1 °C in infected frogs and 36.2 °C ± 1.4 °C to 38.5 °C ± 1.2 °C in uninfected frogs (Table 2). Likewise, mean temperature at loss of righting ability ranged from 37.4 °C ± 2.2 °C to 39.9 °C ± 1.3 °C in infected frogs and 39.6 °C ± 0.5 °C to 40.5 °C ± 1.0 °C in uninfected frogs (Table 2; Fig. 1).

**Table 1:** Summary of analyses of covariance on the effects of *Batrachochytrium dendrobatidis* infection status, infection intensity, elevation (high [15 °C] vs. low [18 °C] acclimation treatments), heat exposure (pulse [26 °C or 29 °C for four hours per day] vs. constant acclimation treatments) and the interactions between infection and acclimation on two metrics of the critical thermal maximum (temperature at onset of spasms and temperature at loss of righting response) for the model amphibian host *Litoria spenceri*, with frog snout-urostyle length as a covariate.

Response	Predictor	Infection status				Infection intensity			
		Sum of Squares	DF	F-value	P-value	Sum of Squares	DF	F-value	P-value
Onset of spasms	Snout-urostyle length	20.59	1	4.382	**0.041**	8.523	1	1.309	0.261
	Infection	70.08	1	14.912	**<0.001**	0.349	1	0.0536	0.818
	Elevation	1.08	1	0.2291	0.634	0.042	1	0.0065	0.936
	Heat	3.05	1	0.6482	0.424	8.649	1	1.3286	0.258
	Infection × elevation	1.88	1	0.4000	0.530	0.077	1	0.0119	0.914
	Infection × heat	16.27	1	3.461	0.068	7.074	1	1.0866	0.305
	Residuals	244.38	52			208.312	32		
Loss of righting	Snout-urostyle length	6.46	1	3.689	0.060	6.874	1	3.2534	0.081
	Infection	12.75	1	7.2832	**0.009**	1.089	1	0.5153	0.478
	Elevation	0.02	1	0.0118	0.914	3.257	1	1.5415	0.223
	Heat	4.25	1	2.4268	0.125	1.227	1	0.5807	0.452
	Infection × elevation	4.51	1	2.5762	0.115	4.754	1	2.2500	0.143
	Infection × heat	11.29	1	6.446	**0.014**	3.173	1	1.5015	0.229
	Residuals	91.06	52			67.614	32

**Table 2:** Average critical thermal maxima for the model amphibian host *Litoria spenceri* with and without infections by the fungus *Batrachochytrium dendrobatidis* and average infection intensities of the infected individuals.

		Infection intensity (zoospore genome equivalents)		Temperature at onset of spasms (°C)		Temperature at loss of righting (°C)	
Elevation	Temperature regime	Mean ± SD for day 36 (sample size)	Mean ± SD for all frogs (sample size)	Mean ± SD in infected (sample size)	Mean ± SD in control (sample size)	Mean ± SD in infected (sample size)	Mean ± SD in control (sample size)
High (15 °C)	Constant	101,267 ± 161,587 (6)	67,402 ± 133,720 (11)	34.2 ± 2.1 (11)	37.9 ± 1.7 (5)	37.4 ± 2.2 (11)	40.1 ± 0.8 (5)
	Pulse (26 °C)	18,113 ± 11,568 (6)	28,486 ± 34,567 (11)	35.3 ± 2.6 (11)	38.0 ± 0.9 (5)	39.5 ± 0.9 (11)	40.5 ± 1.0 (5)
Low (18 °C)	Constant	187,267 ± 307,555 (6)	123,031 ± 244, 297 (10)	34.6 ± 2.5 (10)	38.5 ± 1.2 (5)	38.4 ± 1.4 (10)	39.8 ± 1.3 (5)
	Pulse (29 °C)	8,853 ± 13,121 (6)	9,121 ± 11,999 (7)	35.6 ± 3.1 (7)	36.2 ± 1.4 (5)	39.9 ± 1.3 (7)	39.6 ± 0.5 (5)

The magnitude of the effect of infection status on temperature at loss of righting ability depended on acclimation to heat pulses (p = 0.014; Table 1); compared to uninfected individuals, the temperature at loss of righting for infected individuals under constant acclimation regimes was reduced by an average of up to 2.7 °C, whereas the temperature at loss of righting for infected individuals under pulsed acclimation regimes was only reduced by an average of up to 1 °C (Table 2; Fig. 1). A similar pattern emerged for the magnitude of the effect of infection status on temperature at onset of spasms, although this was not statistically significant. Specifically, the temperature at onset of spasms for infected individuals under constant acclimation regimes was reduced by an average of up to 3.9 °C, whereas the temperature at onset of spasms for infected individuals under pulsed acclimation regimes was only reduced by an average of up to 2.7 °C (Table 2; Fig. 1).

**Figure 2:**
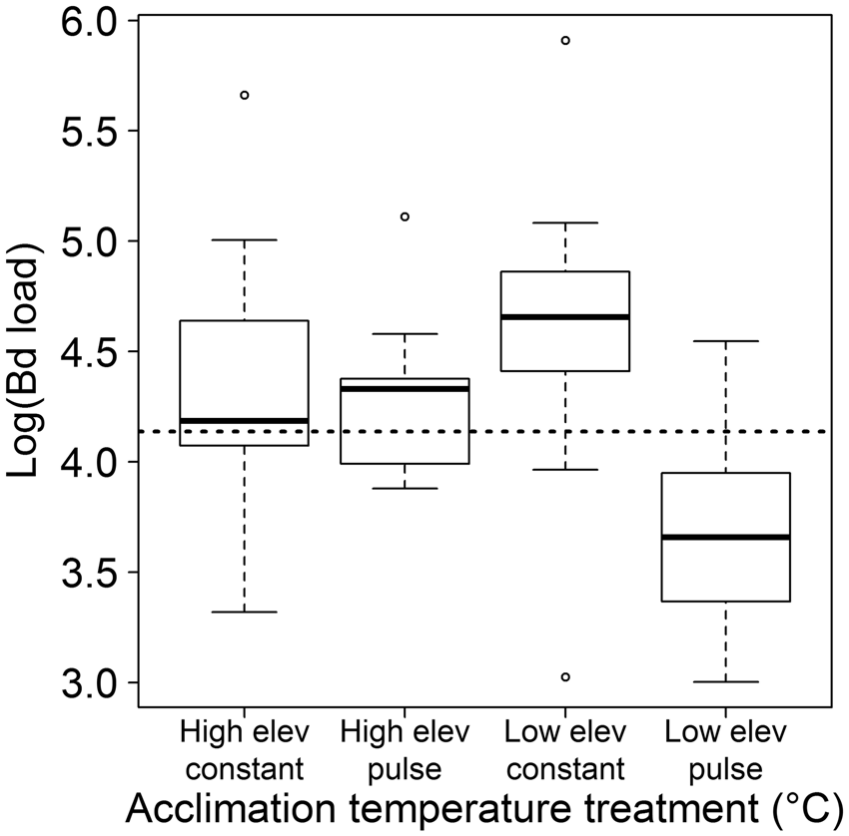
*Batrachochytrium dendrobatidis* infection intensities at the time of measuring the critical thermal maxima of infected *Litoria spenceri* acclimated to four temperature treatments. Dashed line indicates an infection intensity threshold above which frogs were estimated to be at high risk for morbidity and/or mortality from infection.

Infection intensity at the time of CT_max_ measurement varied widely among temperature treatments (Fig. 2). By day 36, the day that we measured CT_max_ in six of the most highly infected frogs from each temperature treatment, the mean infection load exceeded our established threshold for disease development (13,700 ZGE) in all treatments except the low elevation heat pulse treatment (Table 2). After day 36, all frogs from both high elevation treatments and the low elevation constant treatment eventually exceeded the threshold infection intensity. In contrast, only one frog from the low elevation heat pulse treatment exceeded the threshold infection intensity after day 36; the other 10 of 17 frogs in this treatment (59%) maintained low infection loads, eventually cleared their infections, and were therefore excluded from the study. Although infection status had a significant effect on CT_max_, we were unable to detect a statistically significant effect of infection intensity on CT_max_ (Table 1). However, low elevation heat pulse was the only treatment in which (1) the negative effect of infection on CT_max_ was greatly reduced (standard errors of mean CT_max_ temperatures for infected and uninfected frogs overlap (Fig 1) and (2) the average infection loads on day 36 and for the entire duration of the experiment did not exceed the threshold level of 13,700 ZGE (Table 2; Fig. 2).

## Discussion

The global climate and the microclimates experienced by animals are becoming warmer and more extreme^1, 3, 30, 31^, increasing risk of population losses and even species extinctions by decreasing the margin of safety between the maximum heat thresholds of organisms and the maximum ambient temperatures they encounter. For example, recent extirpations of *Sceloporus* lizards were linked to elevated maximum temperatures during the breeding season, which restricted individuals to cool refuges at the expense of foraging and reproduction and in turn caused declining population growth rates since the 1970s^13^. On a shorter-term timescale, record high temperatures on a single day in 2002 were associated with deaths of thousands of flying foxes (*Pteropus* spp.) in eastern Australia^12^. Three years later, and in a neighboring Australian state, another heat wave nearly drove the upland endemic white lemuroid possum (*Hemibelideus lemuroides*) to extinction^32^.

The thermal safety margins of organisms may be compressed not only by rises in environmental temperatures but also reductions in maximum heat tolerance. In our study, heavy fungal infections lowered the critical thermal maxima of juvenile frogs by up to ~4 °C. The maximum heat tolerance of organisms can be determined by temperature effects on the molecules, cells, and biochemical reactions of organ systems, including the circulatory, respiratory, and nervous systems^33–35^. The temperature resistance of these systems could plausibly decrease if already weakened by Bd infection or stress, especially since Bd causes tissue damage^36^ and blocks oxygen, water, and electrolyte balance through the skin^37^. Alternatively, lowered thermal tolerance could indicate manipulation of host physiology by the fungus to promote movement of the host to microhabitats that favor the fungus, which has a low tolerance for elevated temperatures^38, 39^. Further studies are needed to determine the physiological and evolutionary causes of reduced thermal tolerance from infection and the synergistic effects of infection and temperature on fitness at non-critical temperatures.

Ours is the first study to directly test for the effects of a fungal parasite on the upper thermal tolerance of its hosts. Existing literature on the interactions between infection and the upper thermal tolerance of animals is limited to six marine mollusk host species, one freshwater mollusk host species, eight freshwater fish host species, and one amphibian host species (a newt), with trematodes as the dominant parasite (Table 3). Whereas upper thermal tolerance of hosts was enhanced in only 10.5% (2/19) of these host-parasite systems and did not change in 31.5% (6/19) of these systems, our finding that Bd infections lowered host thermal tolerance is consistent with 58% (11/19) of host thermal responses to parasites (Table 3), including the only previous study of thermal thresholds in parasitized amphibians^19^ and similar studies of parasitized fish^20, 21^ and mollusks^22–28^. What does this mean for the present and coming decades, during which animals will face unprecedented changes in the global climate and in rates of infectious disease emergence? Our results suggest that infections by parasites and pathogens may profoundly alter the thermal physiology of hosts, often eliciting significantly reduced heat tolerance. We argue that a diminished upper temperature threshold may not only increase risk of population losses in accordance with the warming tolerance hypothesis^8–11^, but also to perpetuate infections by altering host thermoregulatory behavior, with added implications for host survival.

**Table 3:** Review of studies on the effects of infections on upper thermal tolerance in animal hosts.

Agent phylum	Agent species	Host taxon	Host species	Effect on thermal tolerance	Reference
Arthropoda	*Lernaea cyprinaceae*	freshwater fish	*Pimephales promelas*	no effect	Vaughan and Coble^20^
Arthropoda	*Salmincola edwardsii*	freshwater fish	*Salvelinus fontinalis*	decreased	Vaughan and Coble^20^
Choanozoa	*Ichthyophonus-*like sp.	newt	*Notophthalmus viridescens*	decreased	Sherman^19^
Chytridiomycota	*Batrachochytrium dendrobatidis*	frog	*Litoria spenceri*	decreased	Greenspan *et al*. this study
Platyhelminthes	*Crassiphiala bulboglossa*	freshwater fish	*Perca flavescens*	no effect	Vaughan and Coble^20^
Platyhelminthes	*Cryptocotyle lingua*	marine snail	*Littorina littorea*	decreased	McDaniel^23^
Platyhelminthes	*Himasthla elongata, Renicola roscovita*	marine clam	*Cardium edule*	decreased	Lauckner^27^
Platyhelminthes	*Lepocreadium ovalis*, *Zoogonus rubellus*	marine snail	*Nassarius obsoletus*	decreased	Vernberg and Vernberg^22^
Platyhelminthes	*Maritrema* sp.	marine snail	*Zeacumantus subcarinatus*	increased	Bates *et al*.^28^
Platyhelminthes	*Philophthalmus* sp.	marine snail	*Zeacumantus subcarinatus*	decreased	Bates *et al*.^28^
Platyhelminthes	*Schistosoma mansoni*	freshwater snail	*Biomphalaria glabrata*	decreased	Lee and Cheng^24^
Platyhelminthes	*Uvulifer ambloplitis*	freshwater fish	*Notropis chrysocephalus*	no effect	Hocket and Mundahl^65^
Platyhelminthes	*Uvulifer ambloplitis*	freshwater fish	*Notropis spilopterus*	no effect	Hocket and Mundahl^65^
Platyhelminthes	*Uvulifer ambloplitis*	freshwater fish	*Pimephales notatus*	no effect	Hocket and Mundahl^65^
Platyhelminthes	10 species*	marine snail	*Cerithidea californica*	no effect	Sousa and Gleason^66^
Platyhelminthes	3 species**	marine snail	*Nassarius obsoletus*	increased	Riel^67^
Platyhelminthes (dominant), Acanthocephala, Nematoda	6 species***	freshwater fish	*Lepomis macrochirus*	decreased	Lutterschmidt *et al*.^21^
Platyhelminthes (dominant), Acanthocephala, Nematoda	7 species****	freshwater fish	*Lepomis megalotis*	decreased	Lutterschmidt *et al*.^21^
Platyhelminthes	unknown	marine snail	*Littorina littorea*	decreased	Lauckner^26^
Platyhelminthes	unknown	marine snail	*Nassarius reticulatus*	decreased	Tallmark and Norrgren^25^

*Acanthoparyphium spinulosum, Austrobilharzia sp., *Catatropis johnstoni, Echinoparyphium* sp., *Euhaplorchis californiensis, Himasthla rhigedana, Parorchis acanthus*, unidentified cyathocotylid, unidentified m icrophallid, unidentified renicolid. **Zoogonus lasius, *Himasthla quissetensis*, *Lepocreadium setiferoides*. ***Platyhelminthes: *Neascus* sp., *Proteocephalus* sp.; Nematoda: *Spinitectus carolini*, *Camallanus oxycephalus*, unidentified larvae; Acanthocephala: *Neoechinorhyncus cylindratus*. ****Platyhelminthes: *Crepidostomum cornutum, Neascus* sp., *Proteocephalus* sp.; Nematoda: *Spinitectus carolini*, *Camallanus oxycephalus*, unidentified larvae; Acanthocephala: *Neoechinorhyncus cylindratus*.

**Figure 3:**
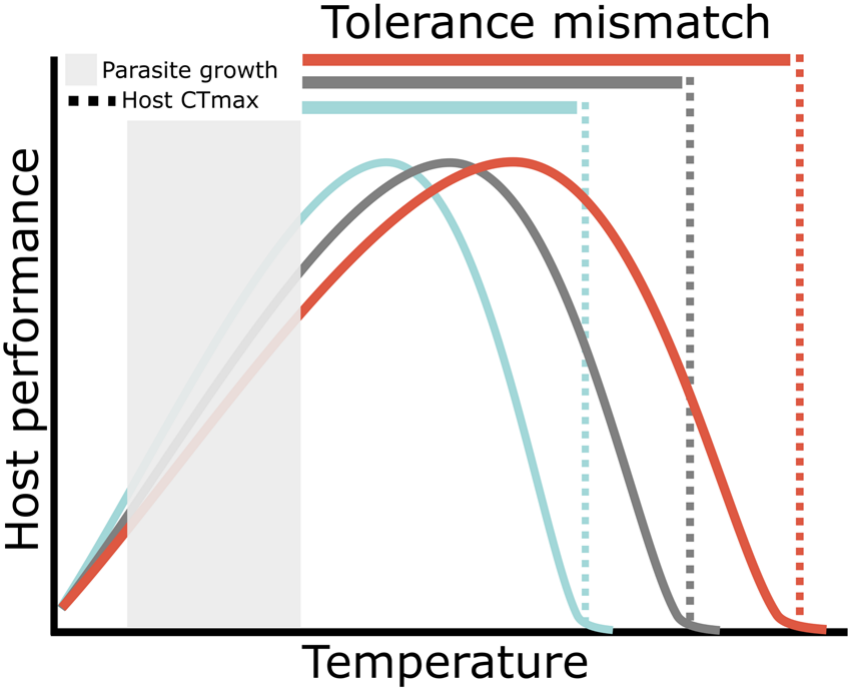
The tolerance mismatch hypothesis predicts that infection risk will decrease as the difference in the thermal tolerance of the host and pathogen (tolerance mismatch) increases^43^. This schematic illustrates the potential effects of parasitic infection on tolerance mismatch for disease systems in which the thermal tolerance of hosts exceeds that of the parasite. Consider a host with a thermal tolerance represented by the gray dotted line. If it becomes infected, its upper thermal tolerance may be reduced (blue dotted line), decreasing tolerance mismatch (blue bar). The host is now more likely to occupy microhabitats (blue performance curve) that are favorable for the parasite, at the expense of protective thermoregulatory behaviors. In rare cases, infections might increase (red dotted line) or have no effect (gray dotted line) on thermal tolerance, thus expanding (red bar) or maintaining (gray bar) the magnitude of thermal mismatch.

In ectothermic hosts, including frogs, behaviors that elevate body temperature may decrease heat-intolerant parasite performance or increase immune function, thereby reducing infection risk or the intensity of existing infections^40–42^. Our study demonstrates the infection-limiting benefits of thermoregulation – for most frogs, four hours of daily exposure to 29 °C (in our low elevation heat pulse treatment) was sufficient to prevent infection levels from exceeding the threshold marking increased risk for morbidity and/or mortality from infection. A recently proposed conceptual model that expands on the relationship between CT_max_ and infection risk predicts that infection risk will increase as the difference between the CT_max_ of the host and parasite decreases (tolerance mismatch hypothesis; Fig. 3)^43^ because infection risk is higher when the host occupies microenvironments that are also favorable for the parasite. Species’ CT_max_ are highly variable even within genera and can be overestimated using laboratory techniques^44, 45^. While our model host species performed at the high end of the CT_max_ spectrum^46^, our study suggests that in ecological systems in which tolerance mismatch is precariously small, high parasite burdens can shrink the gap between host and pathogen thermal tolerances even further (Fig. 3), potentially discouraging protective thermoregulatory behaviors, even at temperatures below upper maxima, and tipping the balance in favor of the parasite.

In contrast to heavy infections, mild infections may not significantly lower host thermal tolerance. The low elevation heat pulse treatment was the only group in which (1) most individuals had infection loads below the threshold for disease development, and (2) the CT_max_ of infected and uninfected individuals was similar, suggesting that any effects of light infection levels on the thermal tolerance of frogs were minimal. However, this warrants further study, especially because we did not detect a statistically significant effect of infection intensity on CT_max_ (Table 1).

We observed higher upper thermal tolerances in infected frogs that were acclimated to realistic daily heat pulses than in infected frogs that were acclimated to constant cool temperatures. These results highlight the importance of incorporating biologically meaningful acclimation temperature regimes into the design of experiments and support the recent finding that small-bodied hosts may be more capable of temperature acclimation than previously thought (Rohr *et al*., in review). Whereas Rohr *et al*. (in review) found that the magnitude of acclimation plasticity may be underestimated in laboratory experiments due to its dependence on acclimation duration and body mass, our use of an atypically long acclimation duration (≥36 days) and small-bodied hosts suggests that our study is robust to these common experimental artifacts. Importantly, however, under most of our acclimation treatments, the magnitude of the parasitism effect exceeded the magnitude of the acclimation effect. This suggests that for populations of some species, even as thermal tolerances are adjusted to long-term increases in temperature from climate change, any benefit this provides to warming tolerance may not be sufficient to protect animals from the thermal consequences of parasitism.

In contrast to infected frogs, which exhibited enhanced thermal tolerances when acclimated to daily heat pulses, we did not detect this acclimation effect in uninfected frogs (i.e., uninfected frogs exhibited similar [or lower, in the case of onset of spasms in the low elevation heat pulse treatment] thermal tolerances when exposed to daily heat pulses compared to constant cool temperatures). It is unclear why the temperature at onset of spasms was reduced in uninfected frogs from the low elevation heat pulse treatment. Lack of an acclimation effect in the other paired constant temperature vs. heat pulse treatments could be attributed to inherent physiological limits (i.e., a ceiling effect) on thermal tolerance or tradeoffs between thermal tolerance and acclimation plasticity^47^. A related avenue for future research is the capacity for heat hardening and resistance adaptation in common parasites.

While gradual increases in average temperatures could favor the hosts of some parasites, such as cool-loving fungi, our study illustrates that we may currently be unable to predict the combined effects of infections and climate change on host populations. Of particular concern are unpredictable heat waves that are long enough to impose thermal stress on hosts but are too short to be therapeutic, for example by ridding hosts of heat-intolerant parasites, or to allow for thermal acclimation. We conclude that infectious disease could lead to increased uncertainty in estimates of species’ vulnerability to climate change.

## Methods

### Acclimation temperature treatments

To generate realistic acclimation temperature treatments, we used body temperature data from *Litoria serrata*, a stream-associated frog of the Australian Wet Tropics^48^. We used temperature-sensitive radio-transmitters (Model A2414; Advanced Telemetry Systems, Isanti, MN) to record the body temperatures of 54 male frogs in rainforests during the dry season (when Bd is typically most prevalent in this region)^48^. The radio-transmitters recorded frog body temperatures every 15 min for 5–11 d. We created simplified, rectangular-wave acclimation temperature treatments to approximate the patterns we found in the field data. We derived the trough temperatures of the rectangular wave treatments from the overall medians of individual median body temperatures at the two high elevation sites (750–800 m elevation; 15 °C) and two low elevation sites (20–100 m elevation; 18 °C) where tracking occurred^48^. We derived the crest temperatures of the rectangular wave treatments from the median of individual maximum body temperatures >25 °C at the same sites (high elevation: 26 °C; low elevation: 29 °C)^48^. We derived the crest length of the rectangular waves from the median of the individual maximum lengths of time that frogs spent with body temperatures >25 °C for all sites combined (4 h)^48^.

**Figure 4:**
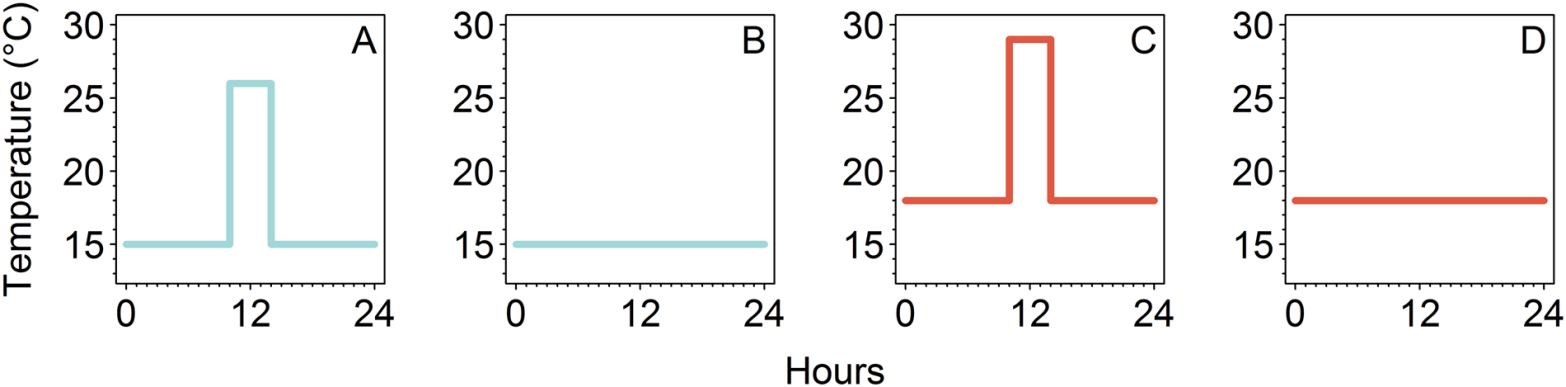
Daily acclimation temperature regimes for experiment investigating the effects of *Batrachochytrium dendrobatidis* infection status, infection intensity, and thermal acclimation on the upper thermal tolerance of the model amphibian host *Litoria spenceri*. (**A**) daily rectangular wave with trough at 15 °C and crest at 26 °C for four hours, (**B**) constant 15 °C, (**C**) daily rectangular wave with trough at 18 °C and crest at 29 °C for four hours, (**D**) constant 18 °C.

Thus, our two high elevation treatments were (1) a daily rectangular wave with trough at 15 °C for 20 h per day and crest at 26 °C for four hours per day (hereafter high elevation heat pulse; inoculated: n = 11; control: n = 5) and (2) a constant 15 °C control treatment (hereafter high elevation constant; inoculated: n = 11; control: n = 5; Fig. 4). Our two low elevation treatments were (1) a daily rectangular wave with trough at 18 °C for 20 hours per day and crest at 29 °C for four hours per day (hereafter low elevation heat pulse; inoculated: n = 17; control: n = 5) and (2) a constant 18 °C control treatment (hereafter low elevation constant; inoculated: n = 10; control: n = 5; Fig. 4). The constant temperature control treatments (15 °C and 18 °C) served as a standard against which to observe effects of acclimation to realistic heat pulses on host thermal tolerance. Our temperature treatments are also pertinent to Bd physiology as this fungus shows optimal short-term growth at 15–25 °C, and ceases growth and reproduction at 26–29 °C^29, 38, 39, 49, 50^.

### *Batrachochytrium dendrobatidis* cultures and inoculations

We used the Bd isolate Paluma-Lseratta-2012-RW-1. This isolate is part of the collection maintained at the College of Public Health, Medical, and Veterinary Sciences, James Cook University. This isolate originated from an adult *L*. *serrata* that was collected from Birthday Creek, a site in the Wet Tropics region of Queensland, Australia (18°58′54″ S, 146°10′02″ E), and died in captivity. The isolate had been cryo-archived after two passages in nutrient broth. We revived an aliquot of the isolate and cultured it in tryptone/gelatin hydrolysate/lactose (TGhL) broth in 25-cm^3^ tissue culture flasks, passaging it twice before the experiment and maintaining cultures at 22 °C.

To obtain zoospores for inoculations, we inoculated Petri dishes containing TGhL broth in 1% agar with ~1/3 ml of cultured broth. Plates were partially dried in a laminar flow cabinet, incubated at 21 °C for four days, and then maintained alternatingly at 4 °C and 21 °C to sustain growth and zoospore production. For each inoculation, we added up to 4 ml of deionized (DI) water to the dishes to form a zoospore suspension. We then combined the liquid contents of each dish, calculated the concentration of zoospores with a hemocytometer (Neubauer Improved Bright-line), and added DI water to produce a final concentration of 1 × 10^6^ zoospores per ml. We prepared a sham (control) inoculant by following the same protocol but with Petri dishes containing only nutrient agar.

All experimental protocols were approved by the James Cook University Animal Ethics Committee in accordance with permit A2234. We experimentally infected captive-reared (sourced from a captive breeding facility at the Amphibian Research Centre, Victoria, Australia), juvenile *Litoria spenceri*. Use of captive-reared frogs ensured no previous exposure to Bd, which can influence the progression of subsequent infections^51^; captive-reared *L. serrata* were unavailable. Our host species basks on streamside rocks and thus could experience similar patterns of temperature variation in its native habitat^52^. Experimental Bd inoculations often yield infection rates < 100% and infection levels are commonly quite variable over time. We therefore used a lower number of sham-inoculated control animals (n = 5 per treatment) than Bd-inoculated treatment animals (n = 10–17 per treatment) to maximize our samples of infected individuals and capture natural variability in infection levels.

We inoculated frogs on three consecutive days. To inoculate, we placed each frog into an individual 70-ml plastic container and added 3 ml of zoospore inoculant or sham inoculant (enough to cover the bottom of the container) to each container using a syringe. We left frogs in inoculant baths for eight hours per day. To ensure regular contact of frogs with the inoculant, we monitored frogs every 15 minutes during each inoculation period. If a frog had climbed out of the inoculant onto the wall of the container, we gently tilted the container to bathe the frog in the inoculant. After each inoculation period, we returned frogs with their inoculant to individual permanent enclosures comprising 70 × 120 × 170 mm plastic containers lined with tap water-saturated paper towel.

We allocated frogs in their individual enclosures to 24 temperature-controlled chambers^53^ on the day after the last inoculation. Six replicate chambers were programmed to execute each of the four acclimation temperature treatments. The chambers were arranged in a blocked design, such that there were six spatial blocks, each containing one chamber following each of the four temperature treatments. The location of each temperature treatment within each block was determined randomly. We distributed inoculated and control frogs into the chambers as evenly as possible and reduced effects of frog history and body size by assigning frogs to temperature treatments proportionally by clutch of origin (reported by captive breeding facility) and snout-urostyle length (measured prior to inoculation). We systematically rotated the placement of the frog enclosures within each chamber every other day to ensure that they were evenly exposed to any local differences in temperature that might exist within the chamber.

### Frog disease monitoring and husbandry

To monitor Bd infection status and intensity, we swabbed frogs upon delivery from the captive breeding facility (all frogs tested negative for Bd before the experiment) and every eight days thereafter following a standard protocol^54^. We determined the number of Bd zoospore genome equivalents (ZGE) per swab with a real-time quantitative PCR protocol modified from Boyle *et al*.^55^.

The temperature-controlled chambers were programmed to maintain a 12 hr: 12 hr light: dark cycle. Every other day, we moistened the paper towels in frog containers with tap water as needed to maintain a consistent moisture level (paper towels were saturated but there was no standing water) and fed frogs pinhead crickets *ad libitum*. We changed paper towels at every other feeding and measured CT_max_ on days on which feeding did not occur.

### CT_max_ measurement and statistical analysis

Our goal was to measure CT_max_ when frogs had well-developed infections but before they displayed clinical signs of infection. By day 36, infection loads in most inoculated frogs were relatively high; in an effort to avoid morbidity and mortality from infection, we measured CT_max_ for a subset of inoculated frogs (n = 6 of the most heavily infected frogs in each acclimation temperature treatment) as well as for all control frogs (n = 5 per temperature treatment) on day 36. We then determined relative risk of morbidity and mortality using a receiver operating characteristic (ROC) analysis for a concurrent experiment with the same cohort of *L. spenceri* ^56^. This analysis indicated that frogs with infection loads >13,700 ZGE had a 63% chance of dying or showing signs of chytridiomycosis. Subsequently, we measured CT_max_ for the remaining inoculated frogs gradually over time, as swab results indicated that frogs were approaching or had exceeded the threshold infection intensity of 13,700 ZGE. We measured CT_max_ within 48 hours of swabbing. All frogs were processed by day 56 except for 10 frogs from the low elevation heat pulse treatment that were excluded from analyses because they never reached the threshold infection intensity and eventually cleared their infections, possibly due to their temperature treatments. All inoculated frogs had sub-clinical Bd infections when we measured CT_max_.

To measure CT_max_, we placed individual frogs into a perforated container containing a suspended thermocouple. Each frog was brought to room temperature in its permanent enclosure and then transferred to the perforated container and placed in a temperature-controlled chamber^53^ programmed to increase from room temperature at a rate of ~1 °C per minute. This rate of temperature increase allows the body temperature of small ectotherms to follow ambient temperature without an appreciable time lag, and is routinely used for measuring CT_max_^57, 58^.

We used two measures of CT_max_^59^: onset of spasms^60^ and loss of righting ability^61^. Onset of spasms, when frogs began displaying erratic movements such as increased jumping and leg twitches, was the first sign of thermal discomfort. We considered this metric to be a conservative estimate of the temperature at which a frog will seek refuge from high temperatures in the wild. After onset of spasms, at each 1 °C increase in chamber temperature, we quickly opened the chamber, gently moved the container until the frog jumped, and closed the chamber. Loss of righting ability, an animal’s upper heat threshold, was determined when animals were unable to right themselves for three seconds after this manipulation. To minimize stress to the frogs, we elected to record the ambient (i.e., thermocouple) temperature at each behavioral indicator of CT_max_ for each frog. Frogs were then immediately placed in room-temperature water to recuperate (all frogs survived). After a recovery period following CT_max_ measurement, we treated Bd infections with Itraconazole^62^.

To determine frog body temperatures at CT_max_, we later exposed four haphazardly selected *L. spenceri* of average sizes to the same program of gradually increasing temperature in the same chamber, following Itraconazole treatment. For each frog, we recorded body temperature at 25 °C, 30 °C, 35 °C, and 40 °C ambient temperature. We measured body temperature with a non-contact infrared thermometer (OS425-LS, Omega Engineering Ltd, Irlam, Manchester, UK; emissivity 0.95)^63^. We then modeled the relationship between ambient and body temperatures using linear regression and used this analysis to convert ambient CT_max_ temperatures to body temperatures for all experimental frogs (y = 0.7985x + 4.0675; R^2^ = 0.9886; 95% confidence interval for slope = 0.751, 0.846; 95% confidence interval for intercept = 2.503, 5.632).

We used R software for all statistical analyses^64^. We used analyses of covariance (ANCOVAs; Anova function in car package; Fox and Weisberg 2011) to test for effects of Bd infection status (infected or uninfected), elevation (high [15 °C] vs. low [18 °C] acclimation treatments), heat exposure (pulse [26 °C or 29 °C] vs. constant acclimation treatments) and interactions between infection status and acclimation on our metrics of CT_max_, with snout-urostyle length as a covariate (n = 69; α = 0.05). To determine whether infection intensity might affect thermal tolerance, we performed separate ANCOVAs using data for infected frogs only, with log-transformed ZGE values as the infection variable (n = 39; α = 0.05).
